# Development and validation of a preoperative nomogram for predicting the surgical difficulty of laparoscopic colectomy for right colon cancer: a retrospective analysis

**DOI:** 10.1097/JS9.0000000000000352

**Published:** 2023-03-31

**Authors:** Ao Yu, Yuekai Li, Haifeng Zhang, Guanbo Hu, Yuetang Zhao, Jinghao Guo, Meng Wei, Wenbin Yu, Zhibo Yan

**Affiliations:** a Department of General Surgery; b Department of Nuclear Medicine; c Department of Gastrointestinal Surgery, Qilu Hospital of Shandong University, Jinan; d Shandong Healthcare Industry Development Group Co. Ltd., Shandong Healthcare, Zaozhuang; e Department of General Surgery, Yutai County People’s Hospital, Jining; f Department of General Surgery, Linyi People’s Hospital, Linyi, People’s Republic of China

**Keywords:** CT value, laparoscopic colectomy, nomogram model, predicting model, right colon cancer, surgical difficulty

## Abstract

In laparoscopic right hemicolectomy for right colon cancer, complete mesocolic excision is a standard procedure that involves extended lymphadenectomy and blood vessel ligation. This study aimed to establish a nomogram to facilitate evaluation of the surgical difficulty of laparoscopic right hemicolectomy based on preoperative parameters.

**Materials and methods:**

The preoperative clinical and computed tomography-related parameters, operative details, and postoperative outcomes were analyzed. The difficulty of laparoscopic colectomy was defined using the scoring grade reported by Escal *et al*. with modifications. Multivariable logistic analysis was performed to identify parameters that increased the surgical difficulty. A preoperative nomogram to predict the surgical difficulty was established and validated.

**Results:**

A total of 418 consecutive patients with right colon cancer who underwent laparoscopic radical resection at a single tertiary medical center between January 2016 and May 2022 were retrospectively enrolled. The patients were randomly assigned to a training data set (*n*=300, 71.8%) and an internal validation data set (*n*=118, 28.2%). Meanwhile, an external validation data set with 150 consecutive eligible patients from another tertiary medical center was collected. In the training data set, 222 patients (74.0%) comprised the non-difficulty group and 78 (26.0%) comprised the difficulty group. Multivariable analysis demonstrated that adipose thickness at the ileocolic vessel drainage area, adipose area at the ileocolic vessel drainage area, adipose density at the ileocolic vessel drainage area, presence of the right colonic artery, presence of type III Henle’s trunk, intra-abdominal adipose area, plasma triglyceride concentration, and tumor diameter at least 5 cm were independent risk factors for surgical difficulty; these factors were included in the nomogram. The nomogram incorporating seven independent predictors showed a high C-index of 0.922 and considerable reliability, accuracy, and net clinical benefit.

**Conclusions:**

The study established and validated a reliable nomogram for predicting the surgical difficulty of laparoscopic colectomy for right colon cancer. The nomogram may assist surgeons in preoperatively evaluating risk and selecting appropriate patients.

## Introduction

HIGHLIGHTS

- Surgical difficulty correlated positively with perioperative mortality and negatively with long-term survival.
- Prediction model of laparoscopic right hemicolectomy to evaluate the surgical difficulty.
- Computed tomography parameters were introduced to establish the prediction model.
- The nomogram incorporating seven independent predictors showed a high C-index.

Since it was first described in 2009^1^, complete mesocolic excision (CME) has become a standard procedure for right colon cancer and is associated with a high lymph node yield and disease-free survival^2^. Compared with the open procedure, laparoscopic right hemicolectomy reportedly has the advantage of faster recovery and comparable long-term results^3^. Thus, laparoscopic right hemicolectomy with CME is widely accepted for colon cancer. However, owing to the complexity of laparoscopic right hemicolectomy, difficult cases have a notable incidence of intraoperative complications (18.0%) and of conversion from laparoscopy to the open approach (6%)^4^. Although conversion and intraoperative complications should not be considered surgical failures, these factors influence the postoperative recovery. Thus, it is extremely important to preoperatively evaluate the surgical difficulty and select appropriate patients in accordance with the surgeons’ experience.

Previous studies have mainly focused on the establishment and validation of nomograms for predicting surgical difficulty in laparoscopic splenectomy and laparoscopic surgery for rectal cancer and sigmoid cancer^5^–^8^. However, the systematic evaluation of the surgical difficulty of laparoscopic right hemicolectomy is very rare.

Preoperative computed tomography (CT) scanning is recommended to determine the optimal therapeutic strategy for patients with colorectal cancer. CT is applied to detect distant metastasis and is also used as a local staging tool that can measure the tumor size and invasion depth with acceptable accuracies and sensitivities^9^. Moreover, the arteries, veins, and blood supplies of the tumor and right colon are visible under CT angiography. As right hemicolectomy with CME involves the identification and ligation of several blood vessels, such as the ileocolic artery, ileocolic vein, right colonic artery, right colonic vein, and Henle’s trunk^10^, the distribution and variations of the blood vessels seen on CT angiography may influence the surgical difficulty.

The aim of the present study was to define preoperative predictors (including CT parameters) that affect the surgical difficulty and to establish a preoperative nomogram for predicting the surgical difficulty of minimally invasive right hemicolectomy, thus facilitating the appropriate selection of patients.

## Materials and methods

### Patients

Data of consecutive patients with right colon cancer who were scheduled for elective radical resection in two tertiary medical centers between January 2016 and May 2022 were retrospectively retrieved from our prospectively collected database and screened for inclusion. The inclusion criteria were: age 18–80 years, with no sex limitation; complete clinical data and follow-up. The exclusion criteria were: preoperative CT image data were incomplete and could not be followed up; T4b cancer or bulky tumors; open surgery; synchronous or double primary cancer. A final total of 568 patients were included. This work has been reported in line with the STROCSS (Strengthening the Reporting of Cohort Studies in Surgery) criteria^11^, Supplemental Digital Content 1, http://links.lww.com/JS9/A198.

### Data collection

For each patient, the following data were extracted from the database:

1. Patient characteristics, namely sex, age, BMI, American Society of Anesthesiologists (ASA) score, smoking status, alcohol consumption status, and comorbidities.
2. Preoperative laboratory results (plasma triglyceride and serum glucose concentrations).
3. Radiological data. Preoperative contrast-enhanced CT scans were conducted using a CT scanner (GE Lightspeed VSX, 64-slice), and the digital data were collected and analyzed by two dependent experienced radiologists who were blinded to the clinical data. The radiologists measured and defined the image parameters, and reanalysis was performed when the differences in results between the two radiologists exceeded 5%. The CT parameters were the adipose thickness at the drainage area of the ileocolic vessels, adipose area at the drainage area of the ileocolic vessels, adipose density at the drainage area of the ileocolic vessels, type of Henle’s trunk, presence of the right colonic artery, abdominal wall adipose area, and intra-abdominal adipose area. There are six types of Henle’s trunk, each of which may affect the difficulty of surgery^12^. Type I: right gastroepiploic vein (RGEV)+anterosuperior pancreaticoduodenal vein (ASPDV)+superior right colic vein (SRCV); type II: RGEV+ASPDV; type III: RGEV+ASPDV+right colic vein (RCV)+superior right colic vein (SRCV); type IV: RGEV+ASPDV+RCV; type V: RGEV+SRCV; type VI: others.
4. Intraoperative data, namely operative time, intraoperative blood loss, and need for conversion.
5. Postoperative data, namely length of hospitalization, morbidities graded using the Clavien–Dindo classification, and perioperative mortality. The 3-year survival rate was analyzed by follow-up.

### Surgical procedure

All procedures were performed by surgeons with experience in more than 100 cases of laparoscopic right hemicolectomy. The whole procedure was performed as previously described^13^.

Briefly, after general anesthesia, the patient was placed in the supine position with their legs spread. The operator stood on the patient’s left side, the first assistant stood on the patient’s right side, and the camera assistant stood between the patient’s legs. Pneumoperitoneum was created with CO_2_ maintained at 12 mmHg, and a 10-mm trocar for laparoscopy was inserted 3 cm below the umbilicus. After meticulous abdominal exploration, four trocars were placed at the left and right sides of the abdomen. The medial approach was commonly applied. The last ileal loop was identified, and the dissection was started behind the pedicle of the ileocolic vessels. Once the correct plane was identified, we switched to dissecting along the superior mesenteric vein (SMV). The ileocolic vessels were clipped at the root and transected with lymph node dissection. The surgical trunk of the SMV was exposed to the level of the inferior edge of the pancreas while being extended in the cephalad direction up to the origin of the right colic artery, if present, and toward the middle colic artery. The Henle’s trunk was dissected and the accessory right colic vein was exposed. The accessory right colic vein and right branches of the middle colic vessels were clipped and divided. After finishing the ligation of the vessels and dissection of the lymph nodes, the proximal transverse and hepatic flexure of the right colon was easily freed and the lateral attachments were freed up, thus completing the right colon mobilization. The anastomosis was accomplished extracorporeally with an end-to-side anastomosis.

### Surgical difficulty criteria and grade

The surgical difficulty criteria were defined as previously described by Escal *et al*.^14^ with modifications. Briefly, five criteria were chosen to evaluate the surgical difficulty and each criterion was assigned a score weighted with coefficients. The total score was 0–10 and the surgical difficulty grade was classified into two levels; a score of less than 3 was taken to indicate a non-difficult procedure, while a score of at least 3 indicated a difficult procedure (Table 1).

**Table 1:** Surgical difficulty grading

	Points
Duration of surgery >240 min	3
Conversion to open procedure	3
Postoperative hospital stay >15 days	2
Blood loss >200 ml	1
Morbidity (grades II and III)	1

### Statistical analysis

The SPSS 25.0 (IBM SPSS Statistics) and R software programs were used for the statistical analysis. The Shapiro–Wilk test and *Q*–*Q* plot were used to determine the normality of continuous variables. The *t* test was used to compare continuous variables, while the *χ* ^2^ test was used to compare categorical variables. Univariable and multivariable logistic analyses were performed to identify preoperative variables that may affect the surgical difficulty and the perioperative mortality. Kaplan–Meier models and log-rank tests were used for the univariable analysis of survival. A machine learning algorithm was applied to determine the best prediction model. A predictive nomogram was developed and validated internally and externally. The Spearman correlation coefficient was used to evaluate the relationship between the surgical difficulty grading score and perioperative outcomes. Receiver operating characteristic curves were used to evaluate the discriminatory power of this scoring system. Statistical significance was defined as *P* < 0.05.

## Results

### Patient characteristics

A total of 568 patients were included in the final analysis. Among the 418 patients from the first medical center, there were 300 and 118 patients assigned to the training data set and internal validation data set, respectively. As shown in Table 2, in the training data set, the median age was 62 years and the median BMI was 24.5 kg/m². The comorbidities were diabetes (43 patients), chronic heart disease (29 patients), and hypertension (101 patients). There were 93 patients with a history of smoking and 62 with a history of alcohol consumption. Thirty-six patients had a high plasma triglyceride concentration (88.0%) and 43 had a high serum glucose concentration (85.7%).

**Table 2:** Patient characteristics

	Training data set (*N*=300)	S–W test, *P*	Internal validation data set (*N*=118)	S–W test, *P*	*P*	External validation data set (*N*=150)	S–W test, *P*
Sex							
Male	187 (62.3%)		71 (60.2%)		0.682	82 (54.7%)	
Female	113 (37.6%)		47 (39.8%)			68 (45.3%)	
Age (mean±SD, years)	62.00±12.13	0.002	60.90±13.27	0.001	0.441	62.32±11.94	0.01
BMI (kg/m^2^)							
≤28	241 (80.3%)	<0.001	95 (80.5%)	0.004	0.724	119 (79.3%)	<0.001
>28	59 (19.7%)		23 (19.5%)			31 (20.7%)	
Smoking							
Yes	93 (31.0%)		35 (29.7%)		0.789	57 (38.0%)	
No	207 (69.0%)		83 (70.3%)			93 (62.0%)	
Alcohol consumption							
Yes	62 (20.7%)		24 (20.3%)		0.941	62 (41.3%)	
No	238 (79.3%)		94 (79.7%)			88 (58.7%)	
ASA							
I	15 (5.0%)		6 (5.1%)		0.953	8 (5.3%)	
II	254 (84.7%)		100 (84.7%)			129 (86.0%)	
III	31 (10.3%)		12 (10.2%)			13 (8.7%)	
Comorbidities							
Diabetes	43 (14.3%)		17 (14.4%)		0.985	35 (23.3%)	
CHD	29 (9.7%)		12 (10.2%)		0.877	22 (14.7%)	
Hypertension	101 (33.7%)		40 (33.9%)		0.964	49 (32.7%)	
Plasma triglycerides							
Normal	264 (88.0%)		103 (87.3%)		0.842	122 (81.3%)	
High	36 (12.0%)		15 (12.7%)			28 (18.6%)	
Serum glucose							
Normal	257 (85.7%)		101 (85.6%)		0.985	114 (76.0%)	
High	43 (14.3%)		17 (14.4%)			36 (24.0%)	

ASA, American Society of Anesthesiologists; CHD, coronary heart disease; S–W, Shapiro–Wilk test.

An overview of the radiological results is shown in Table 3. The median adipose thickness, area, and density values in the drainage area of the ileocolic vessels were 4.06 mm, 1.73 mm^2^, and −60.30 HU, respectively. The right colonic artery was present in 113 patients. One hundred ten patients (36.7%) had a tumor with a diameter at least 5 cm.

**Table 3:** Radiological results

	Training data set (*N*=300)		Internal validation data set (*N*=118)			External validation data set (*N*=150)	
	Value	S–W test, *P*	Value	S–W test, *P*	*P*	Value	S–W test, *P*
Abdominal wall adipose area (mm^2^)	169.74±76.68	<0.001	104.38±49.75	0.702	0.673	99.49±52.34	0.200
Intra-abdominal adipose area (mm^2^)	104.37±47.03	0.014	166.26±72.94	0.040	0.990	169.31±77.27	0.107
Adipose thickness at the drainage area of the ileocolic vessels (mm)	4.06±1.37	0.125	4.11±1.35	0.055	0.735	4.14±1.34	0.052
Adipose area at the drainage area of the ileocolic vessels (mm^2^)	1.73±0.94	<0.001	1.95±1.01	0.005	0.565	1.98±0.99	0.016
Adipose density at the drainage area of the ileocolic vessels (HU)	−60.30±31.87	0.009	−60.49±28.45	<0.001	0.954	−59.69±30.05	0.011
Presence of the right colonic artery							
Yes	113 (37.7%)		37 (31.4%)		0.227	46 (30.6%)	
No	187 (62.3%)		81 (68.6%)			104 (69.4%)	
Type of Henle’s trunk							
I	119 (39.7%)		39 (33.0%)		0.194	52 (34.7%)	
II	84 (28.0%)		38 (32.2%)			45 (30.0%)	
III	43 (14.3%)		23 (19.5%)			29 (19.3%)	
IV	14 (4.7%)		6 (5.1%)			10 (6.6%)	
V	40 (13.3%)		12 (10.2%)			14 (9.3%)	
VI	0		0			0	
Ileocolic artery and SMV position							
The artery is in front of the vein	200 (66.7%)		82 (69.5%)		0.579	105 (70.0%)	
The vein is in front of the artery	100 (33.3%)		36 (30.5%)			45 (30.0%)	
Tumor position							
Cecum	86 (28.7%)		32 (27.1%)		0.752	37 (24.7%)	
Ascending colon	214 (71.3%)		86 (72.9%)			113 (75.3%)	
Tumor diameter							
≥5 cm	110 (36.7%)		45 (38.1%)		0.780	58 (38.6%)	
<5 cm	190 (63.3%)		73 (61.9%)			92 (61.4%)	

SMV, superior mesenteric vein; S–W, Shapiro–Wilk test.

The relevant baseline data and an overview of the radiological results in the internal validation data set and external validation data set are presented in Tables 2 and 3. There were no significant differences in baseline data between the training and internal validation data sets (*P*>0.05).

### Intraoperative and postoperative outcomes

As shown in Table 4, in the training data set, 222 (74.0%) patients were classified as the non-difficulty group and 78 (26.0%) were classified as the difficulty group. The operative time, conversion rate, and duration of postoperative hospitalization were increased in the difficulty group compared with the non-difficulty group.

**Table 4:** Intraoperative and postoperative outcome

	Training data set			Internal validation data set			External validation data set		
	Surgical difficulty			Surgical difficulty			Surgical difficulty		
	Non-difficulty (*n*=222)	Difficulty (*n*=78)	*P*	Non-difficulty (*n*=82)	Difficulty (*n*=36)	*P*	Non-difficulty (*n*=104)	Difficulty (*n*=46)	*P*
Operative time (min)	181.26±29.23	258.74±42.13	<0.01	180.24±29.14	266.11±30.41	<0.01	198.99±46.62	237.72±50.59	<0.01
Estimated blood loss (ml)	60.77±28.43	68.21±33.57	0.059	66.22±36.64	59.72±26.99	0.341	64.90±33.99	65.00±33.91	0.984
Conversion to open procedure	0 (0)	6 (7.7%)	<0.01	0 (0)	2 (5.6%)	0.237	0 (0)	3 (6.5%)	0.009
Postoperative hospital stay (days)	8.60±2.95	11.19±4.90	<0.01	9.99±4.41	9.03±3.00	<0.01	9.65±4.14	10.52±3.02	0.022
Postoperative morbidity									
II/III	9 (4.1%)	5 (6.4%)	0.094	6 (7.3%)	3 (8.3%)	0.307	8 (7.7%)	4 (8.7%)	0.835
N stage									
N0	146 (65.8%)	46 (59.0%)	0.521	55 (67.1%)	20 (55.6%)	0.227	75 (72.1%)	20 (43.5%)	0.001
N1	47 (21.2%)	21 (26.9%)		16 (19.5%)	9 (25.0%)		18 (17.3%)	16 (34.8%)	
N2	29 (13.0%)	11 (14.1%)		11 (13.4%)	7 (19.4%)		11 (10.6%)	10 (21.7%)	
T stage									
T1	20 (9.0%)	2 (2.5%)	0.106	8 (9.7%)	1 (2.8%)	0.598	9 (8.7%)	1 (2.2%)	0.336
T2	24 (10.8%)	7 (9.0%)		9 (11.0%)	3 (8.3%)		12 (11.5%)	3 (6.5%)	
T3	150 (67.6%)	63 (80.8%)		56 (68.3%)	30 (83.3%)		74 (71.2%)	40 (87.0%)	
T4	28 (12.6%)	6 (7.7%)		9 (11.0%)	2 (5.6%)		9 (8.7%)	2 (4.3%)	

In the internal validation data set, 82 (69.5%) patients were classified as the non-difficulty group and 36 (30.5%) were classified as the difficulty group. Compared with the non-difficulty group, the difficulty group had a significantly longer operative time (*P*<0.01), and longer duration of hospitalization (*P*<0.01) (Table 4).

In the external validation data set, 104 patients and 46 patients were assigned into non-difficulty and difficulty group, respectively. As shown in Table 4, a significantly longer operative time, higher conversion rate, and longer duration of postoperative hospitalization were observed in the difficulty group.

Meanwhile, univariable and multivariable analyses showed that surgical difficulty was the independent risk factor for perioperative mortality (Table S1, Supplemental Digital Content 2, http://links.lww.com/JS9/A313). And as shown in Figure S1a, Supplemental Digital Content 7, http://links.lww.com/JS9/A318; S1b, Supplemental Digital Content 4, http://links.lww.com/JS9/A315; S1c, Supplemental Digital Content 5, http://links.lww.com/JS9/A316; S1d, Supplemental Digital Content 6, http://links.lww.com/JS9/A317; patients in difficulty groups showed worse prognoses in terms of 3-year survival rate.

### Results of the univariable and multivariable logistic regression analyses

All continuous data sets in the analysis were normally distributed or approximately normally distributed (Tables 2 and 3). Univariable and multivariable analyses showed that the independent risk factors for difficult laparoscopic right hemicolectomy were the adipose thickness at the drainage area of the ileocolic vessels [odds ratio (OR): 1.712, 95% CI: 1.214–2.421], adipose area at the drainage area of the ileocolic vessels (OR: 1.870, 95% CI: 1.102–3.188), adipose density at the drainage area of the ileocolic vessels (OR: 0.983, 95% CI: 0.968–0.997), presence of type III Henle’s trunk (OR: 8.088, 95% CI: 2.429–27.173), presence of the right colonic artery (OR: 2.474, 95% CI: 1.063–5.714), intra-abdominal adipose area (OR: 1.002, 95% CI: 0.992–1.011), plasma triglyceride concentration (OR: 2.814, 95% CI: 0.854–9.492), and tumor diameter at least 5 cm (OR: 8.464, 95% CI: 3.557–20.691) (Tables 5 and 6).

**Table 5:** Univariate logistic analysis in the modeling and the validation group

	Training data set (*N*=300)		Internal validation data set (*N*=118)		External validation data set (*N*=150)	
	OR (95% CI)	*P*	OR (95% CI)	*P*	OR (95% CI)	*P*
Sex (male vs. female)	1.194	0.518	1.058	0.890	1.019	0.958
Tumor diameter						
≥5 cm	7.261	<0.001	5.810	<0.001	8.022	<0.001
<5 cm	Ref		Ref		Ref	
Age	0.969	0.005	0.973	0.089	1.003	0.439
BMI	1.091	0.005	1.043	0.369		
Adipose thickness at the drainage area of the ileocolic vessels	2.705	<0.001	2.984	<0.001	3.169	<0.001
Adipose area at the drainage area of the ileocolic vessels	5.583	<0.001	3.691	<0.001	6.649	<0.001
Adipose density at the drainage area of the ileocolic vessels	0.932	<0.001	0.971	<0.001	0.932	<0.001
Presence of type III Henle’s trunk						
Yes	26.327	<0.001	32.917	<0.001	21.722	<0.001
No	Ref		Ref		Ref	
Presence of the right colonic artery						
Yes	9.661	<0.001	16.782	<0.001	29.762	<0.001
No	Ref		Ref		Ref	
Abdominal wall adipose area	1.004	0.023	1.005	0.061	0.998	0.450
Intra-abdominal adipose area	1.012	<0.001	1.002	0.531	0.995	0.171
Plasma triglyceride concentration						
Normal	2.165	0.034	1.622	0.396	1.780	0.252
High	Ref		Ref		Ref	

OR, odds ratio, Ref, reference.

**Table 6:** Multivariate logistic analysis between the modeling group and the validation group

	Training data set (*N*=300)		Internal validation data set (*N*=118)		External validation data set (*N*=150)	
	OR (95% CI)	*P*	OR (95% CI)	*P*	OR (95% CI)	*P*
BMI	1.039 (0.935–1.163)	0.510	1.201 (0.813–1.776)	0.089	1.179 (0.815–1.706)	0.383
Adipose thickness at the drainage area of the ileocolic vessels	1.712 (1.214–2.421)	0.002	3.155 (0.978–10.183)	0.055	3.124 (1.002–9.745)	0.050
Adipose area at the drainage area of the ileocolic vessels	1.870 (1.102–3.188)	0.021	19.032 (2.540–142.628)	0.004	23.143 (2.976–179.963)	0.003
Adipose density at the drainage area of the ileocolic vessels	0.983 (0.968–0.997)	0.018	0.937 (0.885–0.992)	0.025	0.926 (0.874–0.982)	0.010
Presence of type III Henle’s trunk						
Yes	8.088 (2.429–27.173)	0.001	234.20 (2.976–18 431.63)	0.014	186.69 (2.341–14 885.4)	0.019
No	Ref		Ref		Ref	
Presence of the right colonic artery						
Yes	2.474 (1.063–5.714)	0.035	12.986 (2.976–18 431.63)	0.043	14.740 (1.183–183.613)	0.037
No	Ref		Ref		Ref	
Intra-abdominal adipose area	1.002 (0.992–1.011)	0.720	0.998 (0.985–1.012)	0.825	0.992 (0.981–1.004)	0.205
Plasma triglyceride concentration						
Normal	2.814 (0.854–9.492)	0.093	1.622 (0.076–52.763)	0.396	3.137 (0.119–82.364)	0.493
High	Ref		Ref		Ref	
Tumor diameter						
≥5 cm	8.464 (3.557–20.691)		327.39 (5.093–21 043.82)		514.06 (8.550–30 907.07)	0.003
<5 cm	Ref	<0.001	Ref	0.006		

OR, odds ratio, Ref, reference.

### Use of a machine learning algorithm for the selection of the optimal prediction model

Four prediction models [logistic regression, support vector machine (SVM), boosting, and random forest] were established by machine learning. The SVM model had the lowest root mean square error (RMSE) (0.675), while the boosting and random forest models had RMSEs of 0.737 and 0.762, respectively; however, the logistic regression model was found to be unsuitable for RMSE evaluation. The area under the receiver operating characteristic curve (AUC) values were 0.823 for the logistic regression model, 0.841 for the SVM model, 0.827 for the boosting model, and 0.832 for the random forest model; the other predictors (accuracy, precision, and recall) are shown in Table 7. There were no significant differences between models in the results of these evaluation methods, which implies that the four models all represent good predictors. Because prediction models can be explained more easily using a nomogram, we chose to establish a logistic regression model in the present study.

**Table 7:** The results of machine learning algorithm.

	Logistic regression	SVM	Random forest	Boosting
RMSE	–	0.675	0.762	0.737
AUC	0.880	0.900	0.893	0.870
Accuracy	0.772	0.826	0.807	0.769
Precision	0.704	0.727	0.708	0.732
Recall	0.720	0.759	0.742	0.728
F1 score	0.824	0.842	0.832	0.827

AUC, area under the receiver operating characteristic curve; RMSE, root mean square error; SVM, support vector machine.

### Development and validation of a predictive nomogram for surgical difficulty

**Figure 1:**
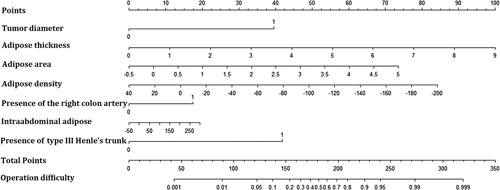
Predictive model of a surgical difficulty nomogram.

**Figure 2:**
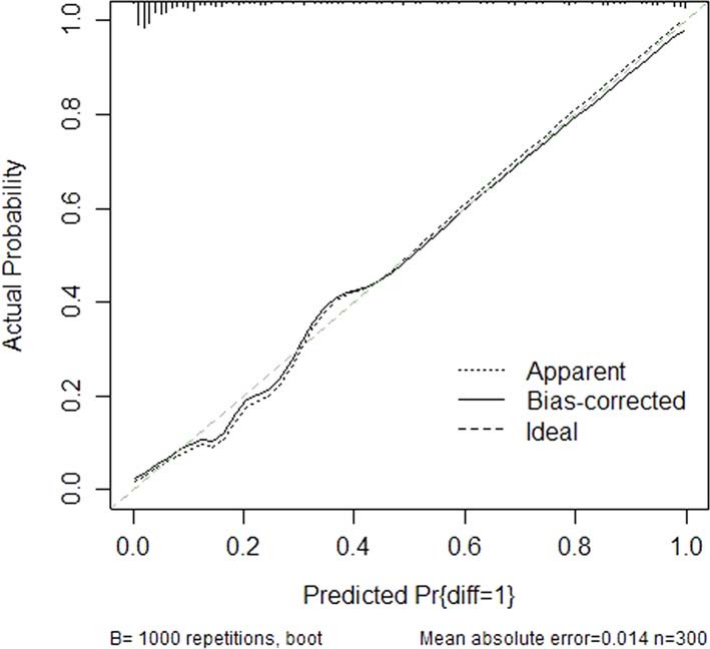
Calibration curve of the nomogram model for the training data set that predicts the risk of surgical difficulty. The diagonal lines represent the perfect prediction of an ideal model; the solid line represents the prediction performance of the prediction model, of which a closer fit to the diagonal dotted line represents a better prediction.

**Figure 3:**
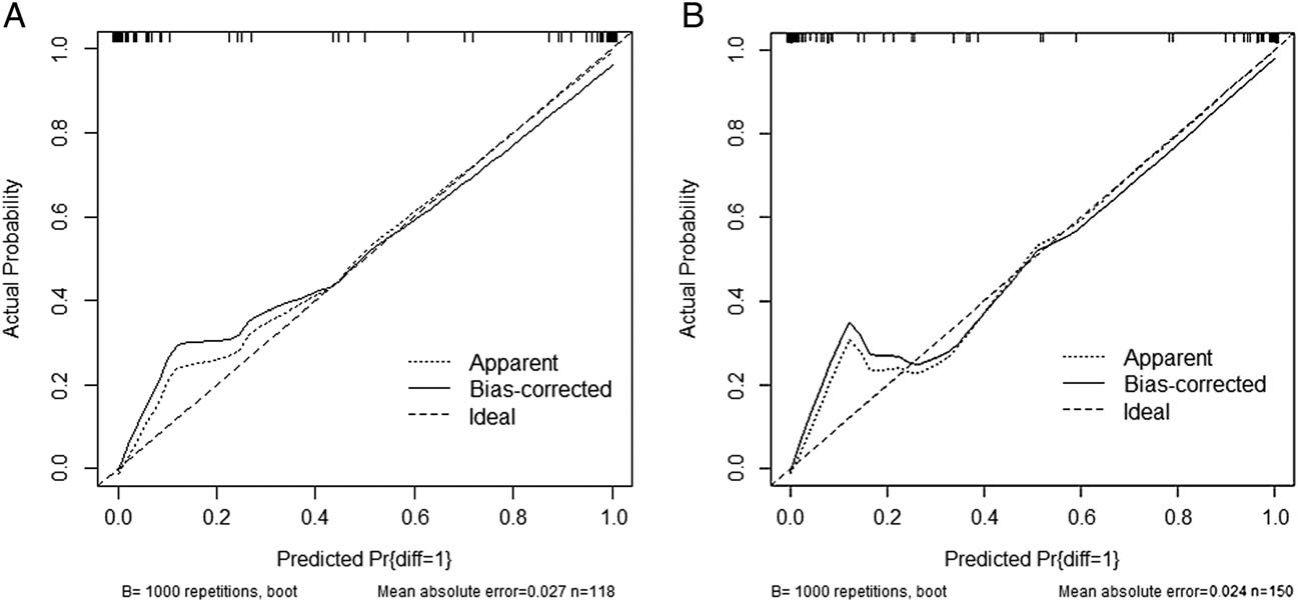
Calibration curve of the nomogram model for internal validation data set (A) and external validation data set (B) that predicts the risk of surgical difficulty.

**Figure 4:**
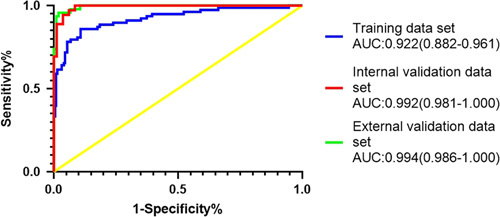
Receiver operating characteristic curves for training data set and validation data sets in predicting surgical difficulty. AUC, area under the receiver operating characteristic curve.

Based on the independent risk factors, a predictive model was established to assess the surgical difficulty of laparoscopic right hemicolectomy (Fig. 1). The calibration curves of the training data set (Fig. 2) and validation data sets (Fig. 3) were highly consistent with the fitting line, which reflects the high accuracy of the prediction model. The C-index of the prediction model was 0.922 (95% CI: 0.882–0.961) for the training data set, 0.992 (95% CI 0.981–1.000) for the internal validation data set, and 0.994 (95% CI: 0.986–1.000) for the external validation data set. The C-indexes were all greater than 0.7, indicating that the prediction ability of the model had high credibility (Fig. 4).

## Discussion

D3 surgery is widely recognized as the standard procedure in laparoscopic radical right hemicolectomy for right colon cancer. Despite multiple extensive discussions about the surgical access for this procedure involving completely medial access by the page-turning approach^13^ and the cephalic approach^15^, these surgical approaches inevitably require total exposure of the SMV. The relatively thin venous wall makes the exposure of the SMV and the management of its branches the most complex part of the D3 procedure. Inexperienced operators are very prone to cause bleeding during the search for the left wall of the SMV or ligation of the vessels, leading to failure of the lumpectomy and conversion to intermediate open surgery. Therefore, the preoperative assessment of the difficulty of SMV exposure and vascular staging are important to appropriately guide the operation. In addition, the size of the tumor and the degree of obesity of the patient alter the complexity of the operation^16^–^18^. Thus, a comprehensive assessment is valuable as a priori knowledge for precise surgery and selection of appropriate patients for laparoscopic surgery.

Considering few studies have assessed the surgical difficulty of the right hemicolectomy, we established a surgical difficulty score system based on the study of Escal *et al*.^14^. In their study, they evaluated the surgical difficulty of rectal cancer based on seven criteria, namely duration of surgery, conversion to open surgery, blood loss, length of hospital stay, morbidity, margin status of the surgical specimen, and the addition of transanal total mesorectal excision to transabdominal surgery. We adopted five of these criteria that suited the surgical characteristics of right hemicolectomy and validated the criteria in our department. Consistent with previous research^19^, we found that increased surgical difficulty was positively correlated with perioperative mortality and negatively correlated with long-term survival, confirming that the criteria selected for the evaluation of surgical difficulty in our study were relatively objective and strongly correlated with the perioperative and long-term survival outcomes.

In the present study, retrospective analyses of 300 patients’ data using a multifactorial approach revealed that the factors influencing the occurrence of surgical difficulty were the adipose thickness, area, and density at the drainage area of the ileocolic vessels, presence of the right colonic artery, presence of type III Henle’s trunk, abdominal adipose area, plasma triglyceride concentration, and tumor diameter at least 5 cm. The BMI was not an influencing factor, which is in contrast to a previous study that reported that BMI influences the difficulty of surgery for colon cancer^20^. Considering the inconsistency of the relationship between the BMI and abdominal visceral fat^21^, the concepts of the adipose CT value, adipose density, adipose volume, and adipose thickness were introduced to more accurately reflect the adipose volume and adipose density in the free region of the right hemicoelomic vessels^22^,^23^. We found no effect of the adipose density of the abdominal wall and adipose volume of the abdominal cavity on the surgical difficulty, while the adipose thickness, area, and density at the drainage area of the ileocolic vessels had a significant effect. Measuring the peri-ileal adipose tissue volume and density achieves a more precise understanding of the difficulty of surgery.

Staging the Henle’s trunk of all patients in the training data set showed that 119 patients had type I (39.7%), 84 had type II (28.0%), 43 had type III (14.3%), 14 had type IV (4.7%), and 40 had type V (13.3%)^24^,^25^. Of these, the difficulty of surgery was significantly increased in patients with type III Henle’s trunk owing to the extended time required to identify and protect its branches, as the presence of more branches increases the risk of iatrogenic injury and bleeding.

Right colonic angiogenesis reportedly occurs in ~12–45% of patients^26^ and was found to be present in 36% of patients in our case series. The right colonic vessel is frequently overlooked due to its relatively low prevalence. Careless dissection may result in unexpected bleeding from the right colonic vessel, making the surgical procedure more difficult. In addition, the blood lipid concentration and blood glucose concentration may influence the adipose density^27^. The blood lipid concentration was revealed as a risk factor for surgical difficulty in the univariable analysis of the present study, while both the blood lipid and glucose concentrations had no effect on the surgical difficulty in the multivariable analysis.

Based on the multivariable analysis results, the optimal model (logistic regression model) was selected by the machine learning algorithm, and the prediction model of surgical difficulty was established. The calibration curve was highly consistent with the fitting line, reflecting the high accuracy of the prediction model. The C-indexes of the prediction model were 0.922 for the training data set, 0.992 for the internal validation data set, and 0.994 for the external validation data set, indicating the high credibility of the prediction capability of the model.

The present study has certain limitations. First, this is a retrospective study with small sample sizes, and larger sample sizes from more centers are warranted for further validation. Second, few studies have evaluated predictive models of the difficulty of colon surgery based on CT values, and many young surgeons lack experience in assessing CT values, which may limit the wide application of our study. Despite these limitations, to our knowledge, the present study is the first to construct a nomogram based on CT values to assess the difficulty of right hemicolectomy, which may contribute to the stratification of patients before D3 radical surgery for colon cancer. Internal validation, external validation, and bootstrap validation in the cohort indicated the good predictive power of the model, which is conducive to making surgical decisions and reducing the incidence of postoperative complications after D3 surgery.

## Ethics statement

The study was approved by the Ethics Committee of Qilu Hospital of Shandong University and conducted in compliance with the Helsinki Declaration (KYLL-202111-087-1). It was registered with the China Clinical Trial Registration Center (No. ChiCTR2200060774). The patients/participants provided their written informed consent to participate in this study. Written informed consent was obtained from the individual(s) for the publication of any potentially identifiable images or data included in this article.

## Sources of funding

The present study was supported by the Shandong Provincial Natural Science Foundation (No. ZR2021MH042), Horizontal Project of Shandong University (No. 6010119075).

## Author contribution

A.Y.: collected the data, analyzed and interpreted the data, writing – original draft, and contributed equally to this work; Y.L.: contributed equally to this work; H.Z., G.H., J.G., M.W., and W.Y.: analyzed and interpreted the data; Y.Z.: collected the data; Z.Y.: conceived and designed the project. All authors read and approved the final manuscript.

## Conflicts of interest disclosure

The authors declare that the research was conducted in the absence of any commercial or financial relationships that could be construed as a potential conflict of interest.

## Research registration unique identifying number (UIN)

1. Name of the registry: China Clinical Trial Registration Center.
2. Unique identifying number or registration ID: No. ChiCTR2200060774.
3. Hyperlink to your specific registration (must be publicly accessible and will be checked): ChiCTR2200060774.

## Guarantor

Zhibo Yan, Department of Gastrointestinal Surgery, Qilu Hospital of Shandong University, Jinan, China, E-mail: zhiboyan@sdu.edu.cn

## Data availability statement

The original contributions presented in the study are included in the article. Further inquiries can be directed to the corresponding author.

## Provenance and peer review

Not commissioned, externally peer-reviewed.

## Supplementary Material
