# Supplementary material for: Development and validation of a preoperative nomogram for predicting the surgical difficulty of laparoscopic colectomy for right colon cancer: a retrospective analysis
Source: Int J Surg. 2023 Mar 31;109(4):870–8. doi: 10.1097/JS9.0000000000000352 (PMC10389525; doi:10.1097/JS9.0000000000000352)
Supplement: Supplementary file 2 [file js9-109-0870-s002.docx]

|  |  | Univariate analysis(N=568) |  | Multivariate analysis(N=568) |  |
| --- | --- | --- | --- | --- | --- |
|  |  | *OR* (95% *CI*) | *P* | *OR* (95% *CI*) | *P* |
| Sex (male vs. female) |  | 1.431 | 0.511 | 0.773 | 0.759 |
| Age |  | 1.019 | 0.890 | 1.028 | 0.313 |
| BMI (kg/m^2^) | ≤28  ＞28 | 1.044  ref | 0.834 | 1.053  ref | 0.951 |
| ASA | I  II  III | 4.816  ref  ref | 0.051 | 2.772  ref  ref | 0.085 |
| Alcohol consumption | Yes  No | 1.337  ref | 0.562 | 16.400  ref | 0.997 |
| Smoking | Yes  No | 1.160  ref | 0.689 | 15.965  ref | 0.997 |
| Surgical difficulty | non | 24.428  ref | <0.001 | 4.772  ref | 0.001 |
|  | difficult | ref |  | ref |  |

**Table S1. Univariate and multivariate analysis of perioperative mortality**
