# Supplementary figures and images for: Development and validation of a preoperative nomogram for predicting the surgical difficulty of laparoscopic colectomy for right colon cancer: a retrospective analysis
Source: Int J Surg. 2023 Mar 31;109(4):870–8. doi: 10.1097/JS9.0000000000000352 (PMC10389525; doi:10.1097/JS9.0000000000000352)

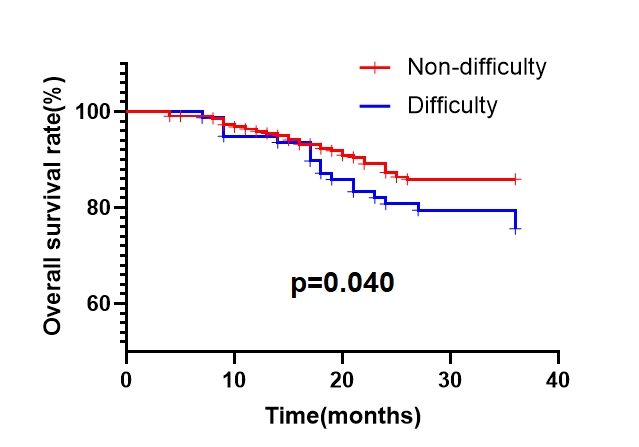

Supplement: Supplementary file 3 [file js9-109-0870-s003.jpg]

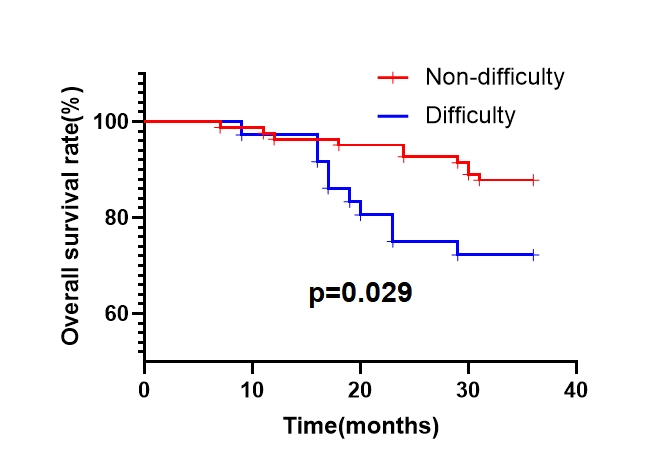

Supplement: Supplementary file 4 [file js9-109-0870-s004.jpg]

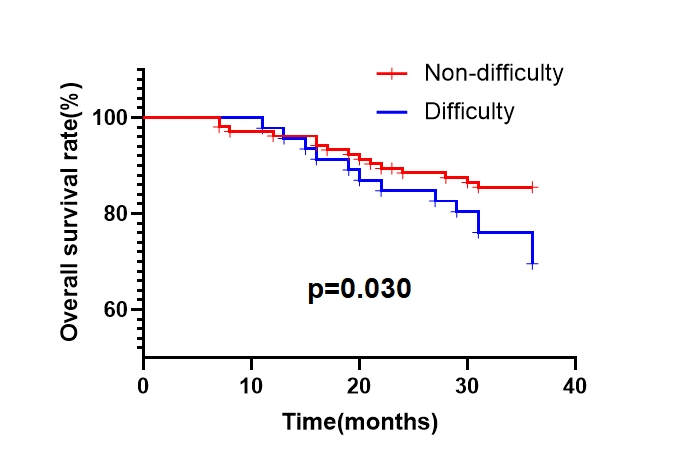

Supplement: Supplementary file 5 [file js9-109-0870-s005.jpg]

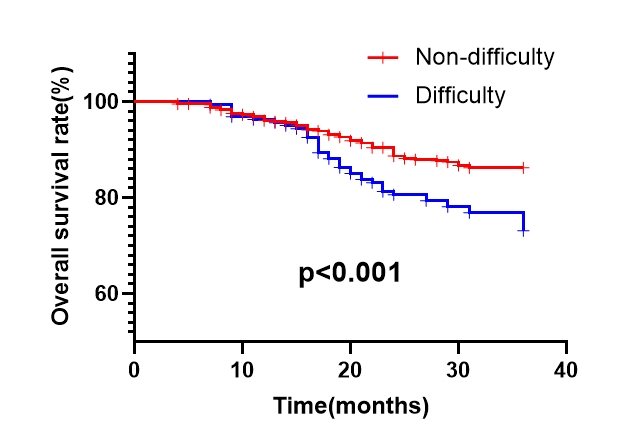

Supplement: Supplementary file 6 [file js9-109-0870-s006.jpg]
